# Novel Scaffold Based on Chitosan Hydrogels/Phthalated Cashew Gum for Supporting Human Dental Pulp Stem Cells

**DOI:** 10.3390/ph16020266

**Published:** 2023-02-10

**Authors:** Yulla Klinger de Carvalho Leite, Antônia Carla de Jesus Oliveira, Patrick Veras Quelemes, Napoleão Martins Argolo Neto, Camila Ernanda Sousa de Carvalho, Huanna Waleska Soares Rodrigues, Michel Muálem de Moraes Alves, Fernando Aécio de Amorim Carvalho, Daniel Dias Rufino Arcanjo, Edson Cavalcanti da Silva-Filho, Alessandra Durazzo, Massimo Lucarini, Maria Acelina Martins de Carvalho, Durcilene Alves da Silva, José Roberto de Souza de Almeida Leite

**Affiliations:** 1 Integrated Nucleus of Morphology and Stem Cell Research (NUPCelt), Federal University of Piaui, UFPI, Teresina 64049-550, PI, Brazil; 2 Research Center on Biodiversity and Biotechnology (BIOTEC), Federal University of Delta of Parnaiba, UFDPar, Parnaiba 64202-020, PI, Brazil; 3 Department of Veterinary Morphophysiology, Federal University of Piaui, UFPI, Teresina 64049-550, PI, Brazil; 4 Laboratory of Antileishmania Activity, Medicinal Plants Research Center, Federal University of Piaui, UFPI, Teresina 64049-550, PI, Brazil; 5 Laboratory of Functional and Molecular Studies in Physiopharmacology (LAFMOL), Federal University of Piaui, UFPI, Teresina 64049-550, PI, Brazil; 6 Interdisciplinary Laboratory for Advanced Materials (LIMAV), Federal University of Piaui, UFPI, Teresina 64049-550, PI, Brazil; 7 CREA-Research Centre for Food and Nutrition, Via Ardeatina 546, 00178 Rome, Italy; 8 Area Morphology, Faculty of Medicine, University of Brasília (UnB), Campus Darcy Ribeiro, Brasília 70910-900, DF, Brazil

**Keywords:** cashew gum, chitosan, chemical modification, scaffold, dental pulp stem cells

## Abstract

Hydrogels are structures that have value for application in the area of tissue engineering because they mimic the extracellular matrix. Naturally obtained polysaccharides, such as chitosan (CH) and cashew gum, are materials with the ability to form polymeric networks due to their physicochemical properties. This research aimed to develop a scaffold based on chitosan and phthalated cashew tree gum and test it as a support for the growth of human mesenchymal stem cells. In this study, phthalation in cashew gum (PCG) was performed by using a solvent-free route. PCG-CH scaffold was developed by polyelectrolyte complexation, and its ability to support adherent stem cell growth was evaluated. The scaffold showed a high swelling rate. The pore sizes of the scaffold were analyzed by scanning electron microscopy. Human dental pulp stem cells (hDPSCs) were isolated, expanded, and characterized for their potential to differentiate into mesenchymal lineages and for their immunophenotypic profile. Isolated mesenchymal stem cells presented fibroblastoid morphology, plastic adhesion capacity, and differentiation in osteogenic, adipogenic, and chondrogenic lineages. Mesenchymal stem cells were cultured in scaffolds to assess cell adhesion and growth. The cells seeded on the scaffold showed typical morphology, attachment, and adequate distribution inside the matrix pores. Thus, cells seeded in the scaffold may improve the osteoinductive and osteoconductive properties of these biomaterials.

## 1. Introduction

Bone tissue engineering is a field that involves the combination of biological constituents with natural materials to create structures capable of contributing to better bone tissue formation and regeneration [1,2]. Materials, such as biopolymers, have been highlighted for presenting a lower risk of rejection when implanted compared to synthetic materials [3,4]. Biopolymers are biocompatible, biodegradable components that tend to accommodate cells as well as promote cell adhesion and proliferation through their porous structure and degrade after new tissue formation [5]. The combined use of cells and scaffold is based on the ability to promote a biomechanical environment suitable for cell adhesion, proliferation, and differentiation [6]. After the initial phase of cell adhesion and proliferation, these cells begin to deposit on the substrate (scaffold) of their own extracellular matrix, stimulating the formation of new tissue [7]. During the process, the scaffold degrades and is continuously eliminated by the body [8]. Human dental pulp stem cells (hDPSCs) have received considerable attention, as they are readily available, and the procedure to obtain them is less invasive [9,10]. The hDPSCs can be differentiated into three mesenchymal cell lineages (osteocytes, chondrocytes, and adipocytes); that is, they are multipotent stem cells and have properties similar to bone marrow stromal cells (BMSCs) [11].

In the context of advances in research, the trend toward the use of biodegradable materials from renewable sources for the preparation of scaffolds has been widely investigated for presenting a bioactive behavior that can mimic the macromolecular environment of the native tissue [12,13]. Chitosan (CH) has characteristics for application in tissue engineering, such as biodegradability, biocompatibility, wide distribution in nature, and similarity with the extracellular matrix [14]. CH, a product of the partial deacetylation of chitin, with a molecular weight between 10 and 1000 kDa, exists mainly in the cell walls of crustaceans, insects, and fungi [15]. It is insoluble in aqueous solutions with a pH above 7. However, acidic solutions (pH < 6) facilitate its solubility [16]. It is a bioactive material that has been widely used in the field of medical materials and biomedicine for its good biological activity, especially as a drug delivery carrier [17,18]. Additionally, due to its versatility related to the degree of deacetylation and variations in molecular weight, chitosan has been widely used in association with other biopolymers, such as silk [19], collagen [20], and agarose [21], in the production of fibrous scaffolds through the electrospinning technique. Chitosan scaffolds produced by electrospinning have been successfully used in studies for neural [22] and bone regeneration [23].

On the other hand, natural polysaccharides have strong hydrogen bonds within and between macromolecules [24]. Furthermore, its stability can be affected by environmental factors (such as temperature and pH). However, the presence of functional groups (-OH) anchored on glycosyl units offers opportunities for chemical modification [25], which makes its use favorable to the construction of hydrogels by gelation mechanisms. Furthermore, it allowed interaction with bioactive ligands without affecting cell behavior [26]. Thus, the cashew gum polysaccharide (CG) has also been used [27,28]. CG is obtained from Anacardium occidentale L. and consists of galactose, arabinose, glucose, uronic acid, and mannose units [29]. It is a biopolymer that has important emulsification and adhesion properties and can function as a stabilizer [30,31]. Its use as an encapsulating agent in delivery systems for pharmaceutical applications [32,33], as well as in the use of edible films for fruit coating [34,35], has been reported. CG has functional groups that can be modified, such as the phthalation reaction [36,37]. Such modification by the insertion of hydrophobic groups in the polymer chain makes CG amphiphilic and highly anionic [38,39]. These characteristics might allow its potential application to produce scaffolds.

Thus, CH has a positively charged amino group, which allows the formation of polyelectrolyte complexes (PEC) in the presence of a negatively charged group, such as the phthalate group inserted in CG [24]. The ionic formation of PEC tends to produce hydrogel-like structures, which enhance the robustness of the scaffold produced. Thus, the present work aims at producing a scaffold based on CH and phthalated cashew gum (PCG) and using it as a three-dimensional support matrix for the growth of mesenchymal stem cells from human dental pulp.

## 2. Results and Discussion

This issue was explored in the following topics: (i) scaffold preparation and characterization; (ii) characterization of human dental pulp mesenchymal stem cells; (iii) cytotoxicity and interference in phagocytic capacity; (iv) stem cells adhesion on the scaffold.

### 2.1. Scaffolds Preparation and Characterization

**Figure 1:**
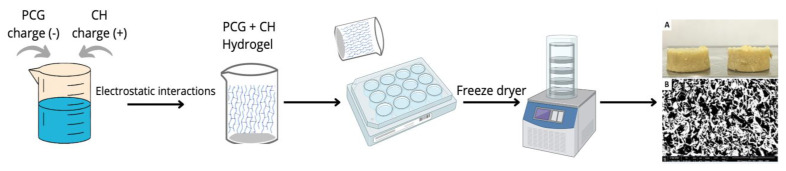
Simplified scaffold production scheme: (**A**) Scaffolds made from chitosan and Cashew gum modified with phthalic anhydride; (**B**) Scanning electron micrograph of a scaffold.

Figure 1 reports the schematic of the scaffold manufacturing process. Three-dimensional porous structures were obtained by lyophilization of PCG/CH hydrogels, prepared using a slow gelling method [40]. After lyophilization, a porous sponge-like structure weighing around 0.036 g, 1.2 cm in diameter, and 0.5 cm in thickness (Figure 1A). When analyzing the pore size of the scaffold by SEM, a structure of micropores and macropores was observed with dimensions ranging from 73 µm to 124 µm and with an average size of 86 µm (Figure 1B). A crucial characteristic of a scaffold is the ability to have interconnected pores, which is favorable for cell adhesion and growth; however, extremely large pores can impair the vascularization of the biomaterial by making difficult contact between cells [41]. Majore et al. [42] reported how the size of mesenchymal cells could vary from 11 to 19 µm and suggested how the size of the pores, presented in the scaffold developed, could be sufficient for cell growth, allowing the exchange of nutrients and metabolites between permeated cells within the material.

**Figure 2:**
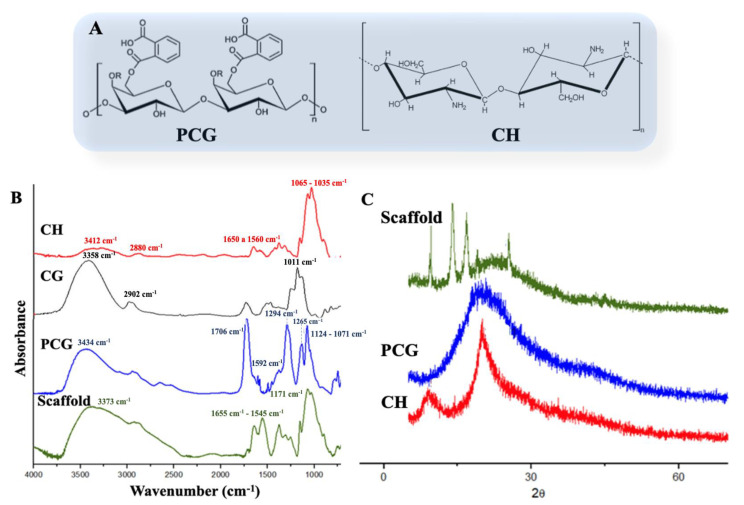
(**A**) Structure of the phthalated cashew gum (PCG) and chitosan (CH) polymers; (**B**) FTIR spectra of CH, Cashew gum (CG), PCG, and Scaffolds; (**C**) X-ray diffraction patterns of scaffold, PCG, and CH.

In this study, as reported in Figure 2A, the scaffold was produced using chitosan and cashew gum modified with phthalic anhydride. The CH FTIR spectrum (Figure 2B) showed a band around 3412 cm^−1^ in relation to the OH axial stretching, superimposed on the N-H stretching band. The band at 2880 cm^−1^ is attributed to the asymmetrical stretching of the C-H group. The spectrum also showed bands from 1650 to 1560 cm^−1^, referring to the presence of amide bands I and II, respectively. Typical bands at 1065 to 1035 cm^−1^ were observed for elongation of C-O in the ether group [43]. Through the phthalate reaction, it was possible to obtain a material with chemical groups that confer anionic characteristics (Figure 2A), as described in Oliveira et al. [36] (2019). The insertion of the phthalate group was confirmed by FTIR analysis, as shown in (Figure 2B). In detail, the PCG spectrum showed a broad band at 3434 cm^−1^ referring to the OH elongation, a band at 1706 cm^−1^ characterizing the presence of carbonyl in the -COOH groups, which represents the modification with phthalic anhydride, and a band at 1294 cm^−1^ referring to C-O elongation. The band at 1265 cm^−1^ can be attributed to the ester group. The bands in the regions from 1124 to 1071 cm^−1^ can be attributed to the alcohol and ether groups of the glycosidic bond, respectively [36]. The FTIR of CG in nature has bands at 3358, 2902, and 1011 cm^−1^, corresponding to the OH of alcohols, C-H, and C-O-C, respectively (Figure 2B) [44].

The scaffold FTIR spectrum (PCG/CH) showed characteristic bands to the precursor polymers, such as the OH band and the glycosidic bonds at 3373 and 1171 cm^−1^, respectively. The scaffold bands that were quite evident are those in the region of 1655 and 1545 cm^−1,^ which, for chitosan, characterizes the NH_3_^+^ groups, while for PCG in the region close to 1592 cm^−1^, it is related to the C=C bonds of the aromatic group. It is evident that the decrease in intensity of the band at 1706 cm^−1^ may indicate that interactions occur between the NH_3_^+^ groups of chitosan and the carbonyl groups of PCG. For scaffold formation, hydrogen bonds may have occurred due to the presence of hydroxyl groups in PCG and CH [28].

The X-ray diffraction technique was used to identify the crystal structure of the polymers and the scaffold (Figure 2C). In the CH diffractogram, the presence of peaks 2θ = 9.3° and 20.1°, respectively, was observed by Braz et al. [45]. PCG showed amorphous characteristics following a certain order of microcrystalline [36]. The scaffold diffractogram (Figure 2C) showed new diffraction peaks at 2θ = 9.6°, 14.0°, 17.08°, 22.36°, 25.4°, and 43.54°. A higher crystallinity was observed in the scaffold with respect to previous reportstudy of Braz et al. [45]. This may be related to the interaction between the two polymers. This phenomenon can also increase the mechanical strength of the scaffold [46].

**Figure 3:**
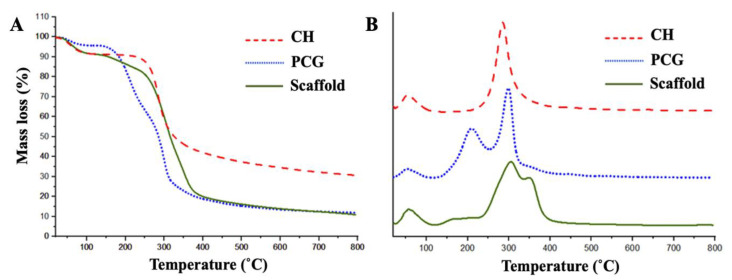
Thermogravimetric curves (**A**) and derivative curves (**B**) of CH, PCG, and scaffolds.

The decomposition profile of the polymers and the scaffold is shown in Figure 3. CH presented two decomposition stages. The first was observed at 52 °C, and the second stage was between 210–391 °C, totaling 60% of lost mass according to Braz et al. [45]. The PCG showed three decomposition stages. The first stage at 52 °C is related to water loss. At 135 °C, the second stage started with 72% mass loss. The third stage occurred near 471 °C with a complete decomposition of the material [37]. The scaffold showed four decomposition stages. The first stage occurred around 56 °C, which is related to the loss of moisture and low molecular weight components that are present in the scaffold structure [37,39]. The second stage was at 138 °C. The third stage was around 300 °C. The fourth stage occurs near 354 °C. Scaffold produced showed a decomposition temperature higher than the polysaccharides used for its manufacture. This fact could be related to the breakage and formation of new connections, providing the scaffold with greater thermal stability. However, Furuya et al. [47], by evaluating the thermogravimetric curve of the chitosan-based scaffold, observed only two mass loss stages; the first stage corresponds to the loss of mass and disassembling the scaffold, and the second stage is like the third stage of loss to mass observed in this study for PCG, approximately at 350 °C.

The swelling rate of the PBS scaffolds was calculated as discussed in the test method section. The scaffold presented a swelling rate equivalent to 96.4%. The balance of hydrophilic and hydrophobic functional groups present in the hydrogel network can result in the retention of large volumes of water in the intermolecular space. As such, the water retention property of hydrogels can produce a moist and biocompatible environment for cell growth, and the bioadhesive of polysaccharides allows them to act as biomimetic scaffolds [40]. In this way, hydrogels are widely used for various tissue engineering applications [48].

### 2.2. Characterization of Human Dental Pulp Mesenchymal Stem Cells

**Figure 4:**
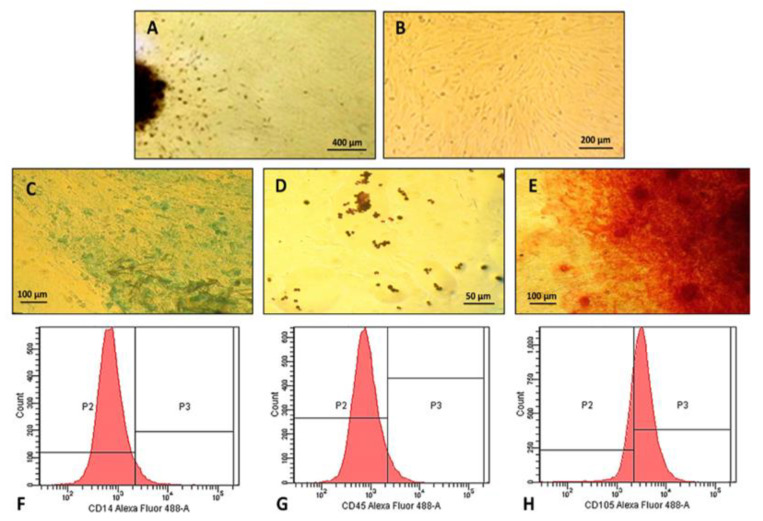
Human dental pulp mesenchymal stem cells characterization: (**A**) Cells in rounded and fusiform format migrating (arrow) from the explant tissue with 10 days of culture (4× objective, bar: 400 µm); (**B**) Cells in first pass; (**C**) Chondrogenic; (**D**) Adipogenic and (**E**) Osteogenic differentiation; (**C**,**E**) 20× objective, bar: 100 µm), (D- 40× Objective, bar: 50 µm). Flow cytometry histograms; (**F**,**G**) Demonstrated the absence of CD45 e CD14 expression and (**H**) CD105 positive expression.

The isolation of hDPSCs was performed using mechanical techniques to preserve the cells from the stress that are subjected to the enzymatic process according to Carvalho et al. [49]. In the first days of cultivation, there were many rounded cells in suspension. The first adherent cells released by the pulp tissue were verified on the 10th day of cell culture (Figure 4A). The establishment of a cell monolayer with a well-defined substrate followed on the 22nd day of cultivation when the culture showed elongated cells arranged in parallel, adopting a typical radial position (Figure 4B).

The isolated stem cells were able to differentiate into bone, adipose, and cartilaginous tissue by induction with specific media, confirmed by the change in morphology and positive staining (Figure 4C–E). In the chondrogenic differentiation (Figure 4C), the change in morphology was observed after staining with Alcian Blue. A strongly stained matrix was identified, demonstrating the multipotentiality of stem cells [50]. In adipogenic differentiation (Figure 4D), there was a change in morphology becoming rounded with cytoplasm containing birefringent fat granules, stained in brown, filling the cytoplasm of the cells [24]. In osteogenic differentiation (Figure 4E), progressive morphological changes were observed from the 10th day of culture. After staining, the culture showed a calcium-rich matrix strongly stained in red and concentrated round spots like osteoblasts [51].

In the immunophenotypic characterization by flow cytometry (Figure 4F,G), the cell population did not express labeling for CD14 and CD45 monoclonal antibodies, excluding the possibility that they were hematopoietic cells [24,52]. The cells showed 59.3% positivity for the CD105 protein, a specific marker of mesenchymal stem cells (Figure 4H) [52]. According to Dominici et al. [53], in addition to the capacity for self-renewal, fusiform morphology, adherence to plastic, and the presence of specific cell surface markers, these cells, to be classified as MSC, must have the ability to differentiate in vitro into adipogenic, chondrogenic and osteogenic cells.

### 2.3. Cytotoxicity and Interference in Phagocytic Capacity

**Figure 5:**
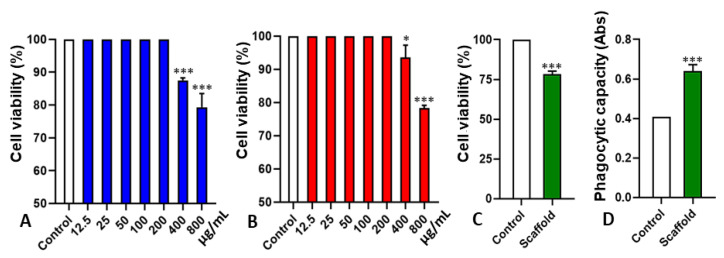
Graphical representation of the cytotoxicity evaluation and macrophages phagocytic capacity determination in PCG, chitosan, and scaffold of different concentrations on stem cells. (**A**) PCG effect on the stem cells viability; (**B**) Chitosan effect on the l stem cells viability; (**C**) Cell viability of stem cells evaluated by the MTT assay after 48 h incubation with developed scaffold; (**D**) Zymosan particles Phagocytosis by macrophages treated with a manufactured scaffold; Statistical analysis following ANOVA One Way, for A and B. *t*-test for C and D; * *p* < 0.05 and *** *p* < 0.001 were considered significant.

The cytotoxicity of the polymers and the scaffold was determined by the MTT assay. As reported in Figure 5A, PCG showed a reduction in cell viability only at the highest concentrations (400 and 800 µg/mL). The work developed by Yamasaki et al. [54] demonstrated that CG did not decrease the cell viability of mouse peritoneal macrophages, both at 24 h and 48 h, suggesting low cytotoxicity at the concentrations tested. Thus, even though the polymer was modified, its toxicity was not altered. A similar result was observed for the CH used to manufacture the scaffold under study (Figure 5B). Cell viability was reduced only at a concentration of 800 µg/mL, and its CC_50_ was above 1000 µg/mL (Figure 5B), thus demonstrating its safety for biological tests. The cytotoxicity evaluation of the scaffold in stem cells showed a small reduction in cell viability in 48 h, not reaching 20% (Figure 5C). The reduction of cell viability in the developed scaffold may have occurred due to the migration of cells into the scaffold. As microscopically demonstrated, the cells already adhered to the bottom of the plate, when in contact with the scaffold, migrated to its interior, interfering with the cell concentration evaluated by the MTT assay of the cells adhered to the plate [46]. The migration of cells into the scaffold pores was also verified by Galdino et al. [55], using scaffolds based on hydroxyapatite–titania, in which they observed a purple coloration of the scaffolds after the MTT assay.

When evaluated in relation to the activation capacity of macrophages, a significant increase could be observed in macrophage activation in the presence of the fabricated scaffold (Figure 5D). With this result, the increase in phagocytic activity of macrophages could be related to the degradability of the scaffold in contact with cells which is a desirable characteristic in polymers and solid devices. It also refers to the fact that when implanted in the system, they undergo degradation by biological particles and cell dispersion [56], forming fragments or other by-products [57]. The study of Bispo et al. [58] shows that hybrid matrices used in tissue regeneration are produced with the potential for degradation in proportion to the regeneration of damaged tissue.

### 2.4. Stem Cells Adhesion on Scaffold

**Figure 6:**
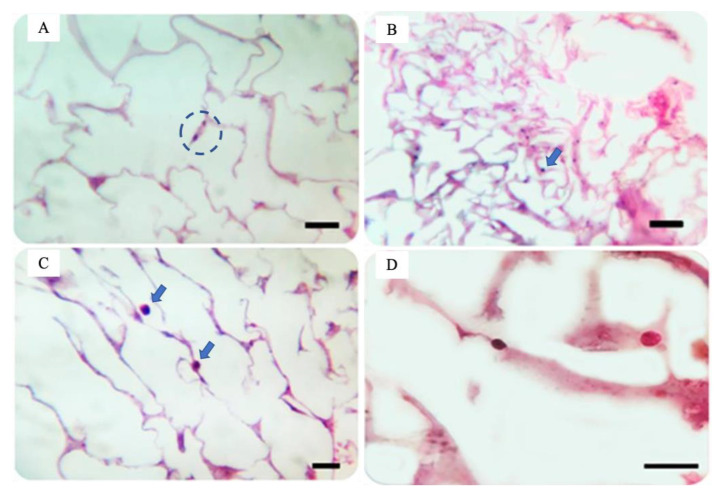
Histological cutting showing the stem cells’ fixation and morphology (main focus) adhered to the scaffold membranes (secondary focus) and scaffold scanning electron micrography (SEM) with stem cells adhered. (**A**) Fusiform cell connected to the matrix membrane (circle) (10× objective, bar: 200 µm); (**B**) Rounded cells adhered to the matrix membrane (arrows) (10× objective, bar: 200 µm); (**C**) Rounded cells adhered and released to the Matrix (arrows) (40× objective, bar: 50 µm); and (**D**): Rounded cells adhered to the matrix (100× objective, bar: 25 µm). Histological plate stained with HE.

The morphological analysis performed by SEM showed how the scaffold developed in this study showed the ability to promote the adhesion of stem cells. The cells grown for seven days in the scaffold showed close contact with the matrix at several points, remaining intact. Through histological sections, it was observed that the cells presented fusiform morphology and fibroblastosis (Figure 6A) are characteristics that are also typical of stem cells [10,53,59]. Cells showed spherical morphology, as reported in Figure 6B–D.

**Figure 7:**
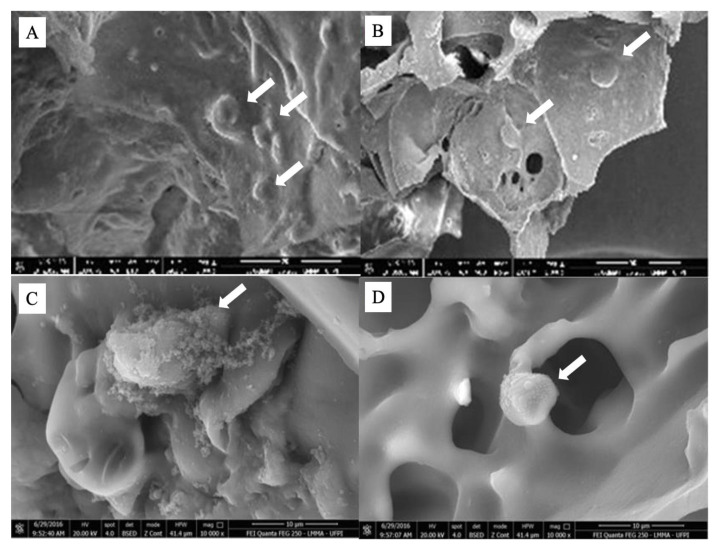
(**A**,**B**) SEM highlighting the stem cells with rounded morphology fixed to the manufactured scaffold (arrows). In (**C**), stem cell displays cytoplasmic extension (arrow); (**D**) Cell surrounded by extracellular matrix; Cell in scaffold pore (arrow). Analysis after 7 days of culture.

Through SEM, a porous surface matrix was also observed, with the presence of fixed cells inside the pores after seven days in culture, with morphology and distribution consistent with the histological description (Figure 7A–D) [24]. These results indicate the efficiency of the developed matrix as a scaffold for cell growth. The size and interconnectivity of the pores were adequate since there was cell adhesion and integration, evidenced by these techniques.

## 3. Materials and Methods

### 3.1. Materials

Dimethylformamide (DMF), analytical grade, was obtained from Dinamica (SP—Brazil). Pure phthalic anhydride, DMEM medium (Dulbecco′s Modified Eagle′s Medium), fetal bovine serum (FBS), penicillin and streptomycin antibiotics, 3-(4,5-100 dimethylthiazol-2-yl) 2,5-diphenyltetrazolium bromide) (MTT), and CH with a medium degree of acetylation (95%) and viscosity of 405 cP was purchased from Sigma–Aldrich (St. Louis, MI, USA). CD14, CD45, and CD105 were obtained from Abcam (Cambridge, MA, USA).

### 3.2. Preparation of Phthalate Cashew Gum

The CG (Mw = 2.12 × 10^4^ g/mol) was obtained from the exudate of A. occidentale L., collected in Parnaiba, Piaui, Brazil. The purification process was carried out according to the method described by de Paula, Heatley and Budd [29]. The cashew tree gum modified with phthalic anhydride (PCG) was modified according to the methodology described by Oliveira et al. [36]. Briefly, 2 g of phthalic anhydride was heated in an oil bath at 130 °C until the solid-to-liquid changed. Then, 1 g of cashew gum was added to the reaction, and after 20 min, the reaction was stopped with 5 mL N,N-dimethylacetamide. The reaction was precipitated, washed in ultrapure water, and dried by lyophilization.

### 3.3. Preparation of Phthalate Cashew Gum/Chitosan Scaffolds (PCG-CH)

The CH and PCG hydrogels were prepared by mixing the CH solution (0.3 g) with PCG (0.3 g) dissolved in acetic acid (0.5%) and distilled water, respectively. After this process, both solutions were mixed under constant stirring for a period of 2 h. The polymer solution was transferred to a 24-well plate and distributed in an amount equivalent to 2.0 mL per well. Subsequently, the plate was taken to the ultra-freezer and frozen at −20 °C for 12 h. Next, the plate was lyophilized for 14 h to obtain porous scaffolds. The scaffolds were weighed on analytical balances. For the biological experiments, the scaffolds were sterilized with ethylene oxide.

### 3.4. Polymers and Scaffold Characterization

#### 3.4.1. Fourier Transform Infrared Spectroscopy (FTIR)

The PCG, CH, and scaffold FTIRs were acquired with a Bomen–Hartmann–Braun spectrometer, model MB series, in KBr tablets, in the range of 4000 to 500 cm^−1^, acquiring the spectrum with 32 scans and a resolution of 4 cm^−1^. A qualitative analysis of the main functional groups was carried out. An assignment of the main bands was carried out by analyzing the acquired spectra and by comparing them with those in the literature.

#### 3.4.2. X-ray Diffraction (XRD)

The crystallographic profile of CH, PCG, and scaffold was determined using a Shimadzu diffractometer, model XR-D600 A. The diffractogram patterns were recorded using randomly oriented assemblies with CuKα radiation in the range 2θ 1.4° to 70°.

#### 3.4.3. Thermal Analysis

Thermogravimetric analysis was performed on TA Instruments SDT Q 600 equipment. Samples of CH, PCG, and scaffold with approximately 8 mg were placed in aluminum crucibles and heated from 10 to 800 °C to 10 °C min^−1^ in a nitrogen atmosphere.

#### 3.4.4. Swelling Behavior of Scaffolds

The scaffold swelling rate was determined by the water absorption percentage. Initially, the dry scaffolds (*n* = 3) were weighed, and their weights were noted (*Wd*). Then, they were immersed in PBS solution with pH 7.4 at 37 °C for 24 h. After 24 h, the moist scaffolds were removed from the PBS solution, and the excess water was rapidly removed with lightly moistened filter paper and then reweighed (*Wm*). The swelling rate was calculated via the equation:

(1) $$Swelling\;\left(\%\right)=\frac{W_{m}-W_{d}}{W_{m}}\times 100$$

#### 3.4.5. Scanning Electron Microscopy (SEM)

The image of the scaffolds was determined by micrographs that were acquired in a scanning electron microscope (SEM) with a field emission gun, brand FEI, model Quanta FEG 250, with accelerating voltage from 1 to 30 kV. To perform the micrographs, the samples were fixed on the aluminum substrate (stub) using double-sided carbon tape. Pore dimensions were determined individually by processing the images using the ImageJ program [60].

### 3.5. Obtaining and Characterization of Human Dental Pulp Mesenchymal Stem Cells

#### 3.5.1. Human Dental Pulp Stem Cells Isolation and Expansion

Six freshly extracted, non-decayed permanent human deciduous teeth were collected for orthodontic purposes (consent of those responsible for the patients through the free and informed consent form approved by the Ethics Committee of the Federal University of Piaui—0218.0.045.000-11). The teeth were submerged in 1 mL of DMEM-F12 culture medium supplemented with 20% fetal bovine serum, 1% penicillin-streptomycin, 1% L-glutamine, and 1% non-essential amino acids and were immediately transferred to the laboratory for pulp isolation.

The isolation of hDPSCs was performed by mechanical digestion [49]. In a laminar flow cabinet, pulp tissue was removed from the teeth with the aid of dental instruments. Then, the tissue was washed 3 times with PBS solution supplemented with 10% antibiotic and submerged in a plate containing DMEM-F12 culture medium supplemented with 20% fetal bovine serum, 1% penicillin-streptomycin, 1% L -glutamine and 1% non-essential amino acids. All pulp tissue was cut into small pieces with a scalpel blade, transferred together with the culture medium to 6-well plates, and subsequently incubated in an oven (TECNAL TE-399^®^) at 37 °C in 5% CO_2_ and 95% humidity. Culture medium changes were performed every 3 days. Upon reaching 80% confluence, the primary cultures were trypsinized with 0.25% trypsin-EDTA solution and incubated at 37 °C for 5 min. After this period, the trypsin action was inactivated by adding twice as much supplemented DMEM-F12 medium. The suspension was transferred to a Falcon tube and centrifuged (FANEM, 280^®^) at 20 °C at 2000 rpm for 10 min. The supernatant was completely discarded, the pellet resuspended in 1 mL of supplemented DMEM-F12, and the first cell count was performed.

The suspended cell content was used for cell expansion. It was plated in 25 cm^2^ tissue culture bottles along with 3 mL of supplemented DMEM-F12 culture medium. They were incubated at 37 °C in 5% CO_2_ and 95% humidity. The cultures were expanded and photographed on an inverted phase-contrast microscope (COLEMAN NIB-100^®^), and subsequent subcultures were performed in an identical manner until the cells reached the 6th passage when they were cryopreserved.

#### 3.5.2. Morphological Characteristics, Adhesion Capacity, and Cellular Plasticity

Adherent and confluent cells with characteristic morphology were observed daily and photographed on an inverted phase contrast microscope. For the analysis of the ability to differentiate into mesenchymal lineages, the cells were plated in a 12-well plate at a density of 5 × 10^3^ cells/cm^2^ and observed until reaching 80% confluence. At the appropriate confluence, chondrogenic, adipogenic, and osteogenic differentiation treatments were initiated.

For chondrogenic differentiation, cells were cultured in DMEM/Hepes medium supplemented with 6.25 μg/mL insulin, 10 ng/mL TGF-β1, and 50 nM ascorbic acid-2 phosphate solution. The medium was changed every 3 days. After 14 days, cells were fixed with 4% paraformaldehyde and stained with Alcian Blue for detection of the extracellular cartilaginous matrix.

For adipogenic differentiation, cells were cultured for 14 days in an IMDM medium containing 20% human plasma, dexamethasone (10^−7^ M), insulin (2.5 μg/mL), indomethacin (5 μM), rosiglitazone (5 μM) and sodium heparin (10 units/mL). Adipocytes were identified through typical morphology by a phase contrast microscope (COLEMAN NIB-100^®^). The culture was fixed with 4% paraformaldehyde and subsequently stained with Oil Red O to identify lipid vacuoles.

For osteogenic differentiation, cells were maintained for 14 days in DMEM/Hepes medium supplemented with fetal bovine serum (10%), dexamethasone (10^−8^ M), ascorbic acid 2-phosphate (5 μg/mL), and β-glycerophosphate (10 mM). The culture was fixed with 4% paraformaldehyde and stained with Alizarin Red dye, detecting differentiation into osteogenic cells.

#### 3.5.3. Immunophenotypic Profile of Cultured Cells

Cells were characterized for the presence of mesenchymal stem cell (MSC) markers. Monoclonal antibodies used were CD14, CD45, and CD105. Sixth passage cells were expanded, trypsinized, and 2.5×10^5^ cells were incubated with 10.0 μL of each antibody for 30 min at room temperature. After incubation, the cells were washed and resuspended in 1.0 mL of PBS. Cells were analyzed using a FACS CANTO II flow cytometer equipped with an argon laser at 488 nm. Data analysis was performed using the Cell Quest software (FACS DIVA).

### 3.6. Biocompatibility Assessment of Polymers and Scaffold

#### 3.6.1. CH, PCG, and Scaffold Cytotoxicity on Stem Cells

The cytotoxic effect of the scaffolds, CH and PCG, was determined by the (3-(4,5-dimethylthiazol-2-yl)-2,5-diphenyltetrazolium bromide) MTT assay. 100.0 µL of supplemented DMEM-F12 medium and about 2×10^3^ MSCs per well were added to separate 96-well plates. These cells were incubated at 37 °C and 5% CO_2_ for 24 h for cell adhesion, followed by two washes with supplemented DMEM-F12 medium to remove cells that did not adhere. Subsequently, 100 μL of DMEM-F12 supplemented with different concentrations of GCF and CH were added separately (6.25, 12.5, 25.0, 50.0, 100.0, 200.0, 400.0, 800.0 μg/mL). The plates were incubated for 48 h, and then 10 μL of MTT diluted in DMEM-F12 medium at 5.0 mg/mL was added. Then, they were incubated for another 4 h in an oven at 37 °C with 5% CO_2_; the supernatant was discarded, and 100.0 μL of DMSO was added to all wells. The plates were placed under stirring for 30 min on a Kline shaker (model AK 0506) at room temperature for a complete dissolution of formazan. Finally, the reading was performed at 550 nm in a Biotek plate reader (model ELx800). The procedure was performed in triplicate, and the results were expressed as a percentage and cytotoxic concentration for 50% of MSCs (CC_50_).

To analyze the cell viability of MSCs in contact with the scaffold, a protocol similar to the previous one was used. In a 96-well plate, 100.0 μL of supplemented DMEM-F12 medium and about 2 × 10^3^ CTMP per well were added. These cells were incubated in an oven for 24 h for cell adhesion. The scaffold fragments were washed with culture medium and added to the wells containing the adhered cells, and kept in an oven for 48 h. After the respective times, the scaffolds were removed together with the culture medium, followed by the addition of MTT, incubation, and reading according to the previously described protocol. The procedure was performed in triplicate.

#### 3.6.2. Macrophages Phagocytic Capacity in the Presence of Scaffold

In this experiment, Wistar rats’ peritoneal cells were used. Animal care and experimental procedures were conducted following the guidelines of the Ethics Committee on Animal Experimentation, the Federal University of Piaui (EAEC-UFPI, report 023/14 and 143/16).

Resident macrophages were collected from the 01 Wistar rat’s peritoneal cavity. The animal was euthanized, and the macrophage removal was performed in a laminar flow hood with the animal fixed on the plate in the dorsal decubitus position, administering 8 mL of sterile phosphate-buffered saline at 4 °C, in the abdominal cavity. The cells were counted in the Neubauer chamber, using the Trypan Blue Dye Exclusion Test, obtaining a minimum of 95% living cells. Again, the cells were counted using Neutral Red to adjust the desired concentration of macrophages (2 × 10^5^ cells/mL).

To assess the phagocytic capacity of macrophages in the scaffold, 2 × 10^5^ peritoneal macrophages were plated per well and incubated. After 48 h of incubation at 37 °C and 5% CO_2_, 10.0 µL of Zymosan solution was added, followed by an additional 30 min of incubation in an oven at 37 °C. After this procedure, 100.0 µL of Baker’s fixative was added to stop the phagocytosis process, and after 30 min, the plate was washed with 0.09% saline to remove Zymosan and neutral red not phagocytosed by macrophages. The supernatant was removed, and 100.0 µL of extraction solution was added. Absorbance was measured at 550 nm in a Biotek microplate reader (model ELx800). The procedure was performed in triplicate.

#### 3.6.3. Evaluation of Stem Cells Adhesion on Scaffold

For fixation and evaluation of stem cell integration into the scaffold, an amount of 1×10^5^ cells/mL was resuspended in 1.5 mL of DMEM-F12 culture medium and plated on the surface of the scaffold in 12-well cell culture plates. The plate containing the scaffolds seeded with the cells was incubated in an oven at 37 °C in 5% CO_2_ and 95% humidity for a period of seven days. After the incubation time, the scaffolds were fixed in 4% formaldehyde after dehydration in ethanol and paraffinization. Cell morphology and adhesion to scaffolds were evaluated by histological processing. Microtome sections of 5 µm were made, stained with hematoxylin and eosin, and visualized under a light microscope. Cell adhesion to the scaffolds was also evaluated by SEM. After incubation, the scaffolds were transferred to Falcon tubes and fixed with 2.5% glutaraldehyde for 24 h. Subsequently, the samples were washed with PBS and dehydrated by dilutions in ethanol (30%, 55%, 70%, 88%, 96%, and 100%). After complete drying of the samples, the scaffolds were metalized with gold and observed in SEM (Quanta FEG 250).

### 3.7. Statistical Analyses

To calculate the CC50 with a confidence limit of 95%, the Probit regression model of the SPSS 13.0 program was used. ANOVA analysis of variance followed by the Bonferroni test was performed using the GraphPad Prism version 5.0 program, taking *p*-value < 0.05 as the maximum level of statistical significance.

## 4. Conclusions

In this study, scaffolds containing phthalated cashew gum and chitosan were produced, and their physicochemical and biological properties were explored. The structural modification of phthalation in cashew gum conferred good properties to interact with chitosan. The semi-crystalline structure can lead to greater stability. The swelling rate can produce a compatible environment for cell adhesion and growth. Thus, the production of the PCG-CH scaffold enabled an improved interaction capacity and support matrix for the growth of mesenchymal stem cells. For future work in a complementary way to the steps of this study, it is necessary to carry out mechanical compression tests in order to know the maximum tension supported by the scaffold since this type of effort is one of the main ones to which the bone is subjected. New biological assays are still needed to visualize cell adhesion to the scaffold over a longer period, as well as tests that assess its degradation potential and, finally, to perform an in vivo study in animals for the application of the scaffold, evaluating its behavior in the body fluid. On the other hand, the developed scaffold showed appropriate properties for applications in tissue engineering for mesenchymal stem cell growth.

## Data Availability

Not applicable.
